# Association of imaging biomarkers and local activation of complement in aqueous humor of patients with early forms of age-related macular degeneration

**DOI:** 10.1007/s00417-020-04910-6

**Published:** 2020-09-02

**Authors:** Vasilena Sitnilska, Philip Enders, Claus Cursiefen, Sascha Fauser, Lebriz Altay

**Affiliations:** 1 Department of Ophthalmology, Faculty of Medicine and University Hospital of Cologne, University of Cologne, Kerpener Str. 62, 50924 Cologne, Germany; 2 F. Hoffmann-La Roche AG, Basel, Switzerland

**Keywords:** Complement, Age-related macular degeneration, Imaging biomarkers, Aqueous humor

## Abstract

**Purpose:**

To investigate a possible correlation between established imaging biomarkers for age-related macular degeneration and local complement system activation, measured in aqueous humor (AH) of patients with early stages of age-related macular degeneration (AMD) and controls.

**Methods:**

This analysis included prospectively acquired AH samples of 106 eyes (35 with early/intermediate AMD, 71 controls). The levels of complement protein 3 (C3), 4 (C4), 5 (C5); activation products of complement factor 3a (C3a) and Ba, C3b/iC3b; complement factors B, D, H, I (CFB, CFD, CFH, CFI); and total protein concentration were analyzed. Quantitative levels of complement factors were correlated to the presence of reticular pseudodrusen (RPD), the presence of hyperreflective foci (HRF), and total drusen volume (DV) graded on imaging by spectral-domain optical coherence tomography and using Spearman’s rank correlation test.

**Results:**

DV correlated with C3b/iC3b (*r* = 0.285; *P* = 0.034), C3a (*r* = 0.200; *P* = 0.047), Ba (*r* = 0.262; *P* = 0.009), and C5 (*r* = 430; *P* = 0.005), and showed a tendency towards correlation with C3a (*r* = 0.198; *P* = 0.057). HRF correlated significantly with C5 (*r* = 0.388; *P* = 0.011) and RPD showed a tendency towards correlation with CFB (*r* = 0.196; *P* = 0.050).

**Conclusion:**

In patients with early AMD, HRF and drusen parameters but not RPD show low to fair levels of correlation with local complement activation in patients’ AH. Better understanding of complement activation could provide some insights into the pathogenesis of AMD. Imaging biomarkers could be useful to identify suitable patients for future clinical trials with complement-modulating therapies.

## Introduction


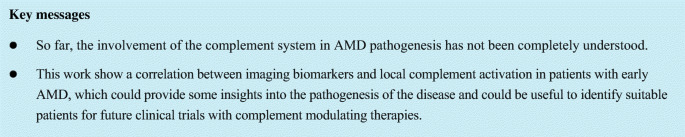
Age-related macular degeneration (AMD) is one of the leading causes for blindness worldwide in elderly people [1]. Since the discovery of complement components in drusen—the hallmark of the disease—the role of complement system in AMD is extensively studied [2–7]. Several genetic studies showed association of genetic variants encoding for complement system components and regulators with increased risk for AMD and its progression [8–12]. The upregulation of complement activation products in serum and plasma of AMD patients further supports the importance of the complement system in AMD. Lately, an increase in systemic complement activation has been associated with the consecutive AMD stages [13–16]. In line with these findings, upregulation of local complement activation products in aqueous humor (AH) has been found in patients with different AMD stages, including even patients with early forms [17, 18]. Although the influence of systemic complement activation on the local inflammation in the eye is not yet completely understood [19, 20], alterations of the complement system seem to occur also in earlier stages of AMD. Their detection can be important for future therapeutic intervention and risk estimation.

With the wide use of non-invasive spectral-domain optical coherence tomography (SD-OCT), distinctive morphological features such as drusen volume (DV), hyperreflective foci (HRF), and reticular pseudodrusen (RPD) were identified as imaging biomarkers for AMD progression [21–25]. It is still unknown whether those imaging biomarkers reflect the level of local complement activation in the eye. Understanding the association between local complement activation and distinct AMD imaging biomarkers, in addition to the individual genetic risk, might contribute to a refinement in patient selection for future clinical trials involving complement-modulating therapies.

To address this important clinical need, this pilot study aimed to investigate the association of DV, presence of HRF and RPD, and local complement activation in AH of patients with early stages of AMD.

## Material and methods

This analysis included clinical data of 106 patients from the Department of Ophthalmology, University of Hospital of Cologne. AH samples were prospectively collected and analyzed for activation levels of complement and protein [18]. Retinal imaging included spectral-domain optical coherence tomography (SD-OCT, Spectralis HRA system; Heidelberg Engineering, Heidelberg, Germany) and digital color fundus photographs (FP, Canon UVI fundus camera; Canon, Tokyo, Japan). Further epidemiological data (age, gender, medical history for hypertension, diabetes, glaucoma, steroid use) were collected.

### Study population

AH samples of patients with early forms of AMD or controls treated in the Department of Ophthalmology, University of Hospital of Cologne, between January 2016 and November 2016, were prospectively collected during routine cataract surgery [18]. In cases where same patients had data of both eyes, one of the eye was randomly selected in order to avoid bias (in total, seven partner eyes were excluded). All patients signed written informed consent; the study was performed in accordance with the tenets of the Declaration of Helsinki and the Medical Research Involving Human Subjects Act (WMO) and was approved by the local ethics committee of the University Hospital in Cologne.

Exclusion criteria were history of systemic/current local steroid use or immunosuppressive therapy and systemic diseases associated with possible complement activation such as autoimmune diseases, infectious diseases, or cancer. Further exclusion criteria were other severe retinal pathologies such as diabetic retinopathy, retinal artery or vein occlusion, macular edema, high myopia (≥6dpt), macular hole, neovascular/geographic AMD or uveitis, as well as insufficient image quality or any ophthalmic surgery in the last 6 months prior to the AH sampling.

### Grading procedure

All patients were graded for the presence of AMD or any other retinal diseases by two graders (VS and LA) using FP and SD-OCT imaging. In the control group, none of the subjects had any drusen or any pigmentary changes in both eyes. Early AMD included the presence of 1–14 medium drusen > 63 μm and ≤ 124 μm in the circular Early Treatment Diabetic Retinopathy Study (ETDRS) grid with or without pigmentary changes or at least ten small drusen (< 63 μm) with pigmentary changes. Intermediate AMD included the presence of any large drusen (≥ 125 μm) or more than 15 medium drusen within ETDRS grid. In the intermediate group, none of the subjects had any geographic atrophy. These criteria are according to the Cologne Image Reading Center and Laboratory (CIRCL) and are similar to the Beckman Initiative for Macular Research Classification [16–18, 26]. Both graders were masked to the complement results at the time of the grading. Discrepancies between graders were resolved by open adjudication.

**Fig. 1:**
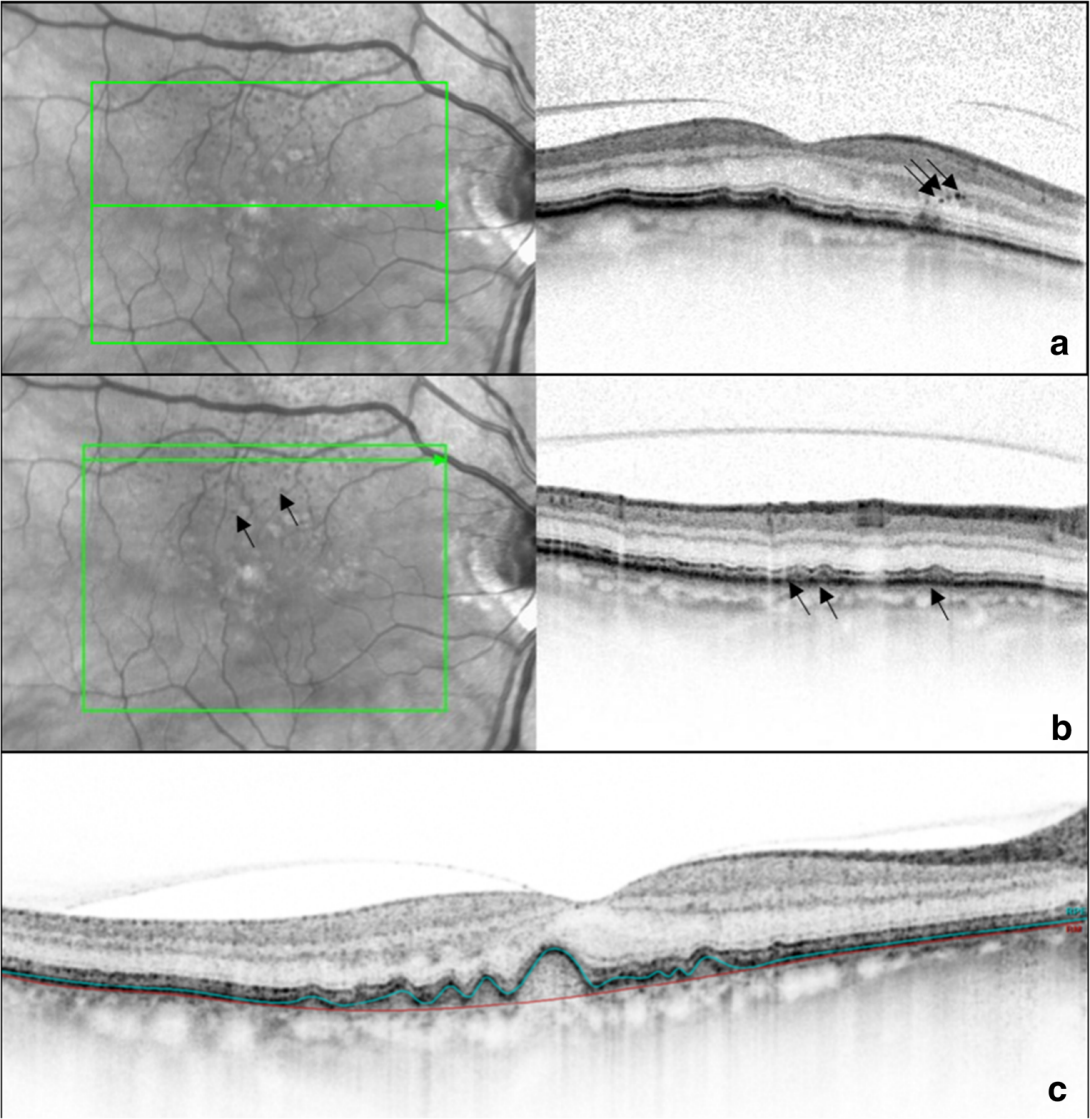
Grading of imaging biomarkers. **a** Hyperreflective foci (black arrows). **b** Reticular pseudodrusen (black arrows). **c** Drusen volume measured as the distance between the Bruch’s membrane (red line) and retinal pigment epithelium (green line)

**Fig. 2:**
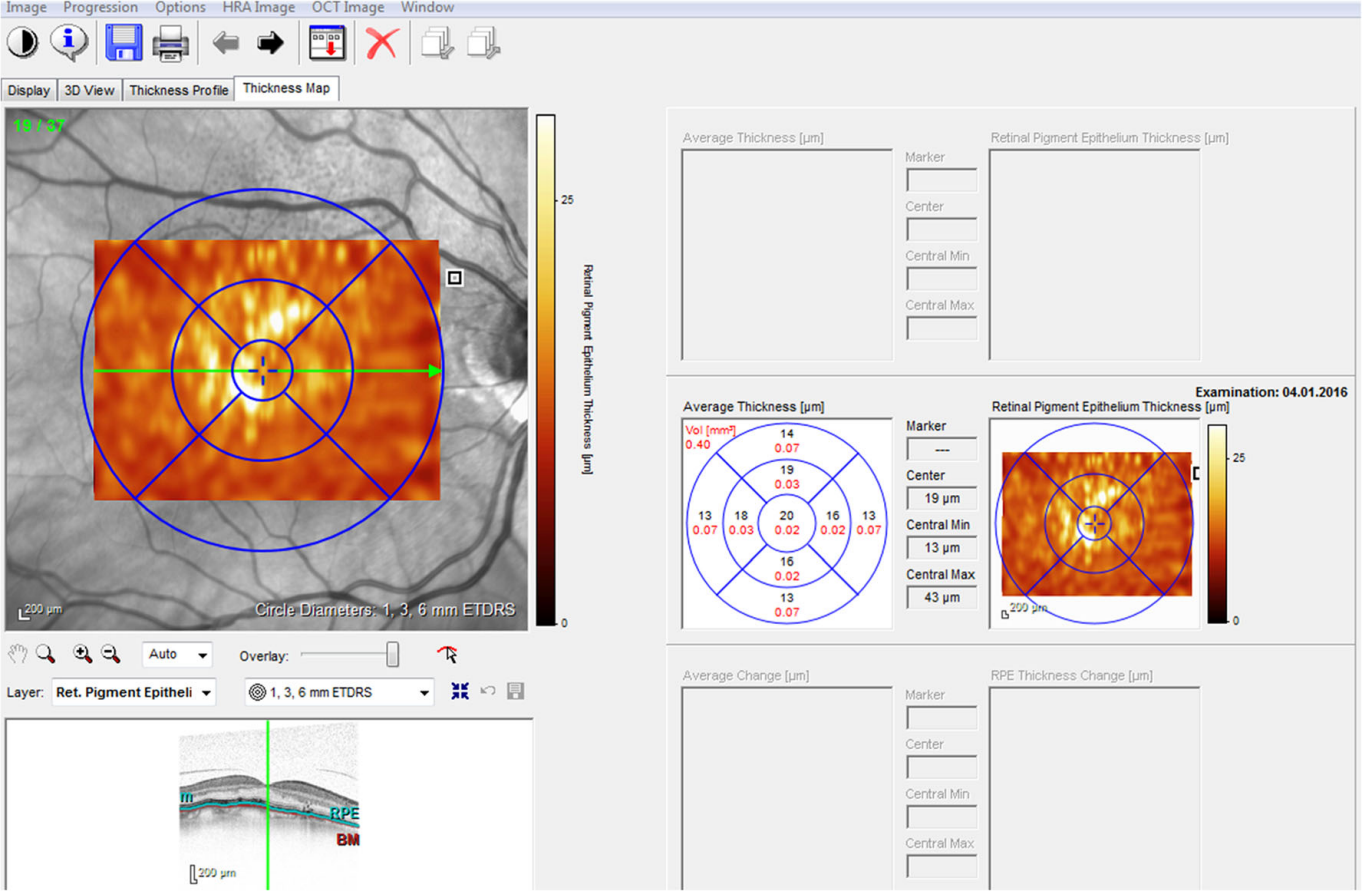
A 6-mm circular ETDRS grid

The following imaging biomarkers were analyzed in SD-OCT: HRF (yes/no in all SD-OCT scans), RPD (yes/no in all SD-OCT scans), and DV (mm^3^) within 6-mm grid placed on all SD-OCT volume scans (Fig. 1). HRF were defined as discrete, highly backscattering lesions within the neurosensory retina with equal or greater reflectivity than the retinal pigment epithelium (RPE) band and HRF was graded as “yes,” if at least three independent significant highly backscattering lesions were found in the outer retinal layers [27]. RPD was defined as subretinal drusenoid deposit and was graded both in SD-OCT and infrared images [28]. DV was calculated using automated retinal layer segmentation provided by Spectralis HRA system, Heidelberg Engineering, Heidelberg, Germany. Drusen contour were measured as the distance between the Bruch’s membrane (BrM) and RPE [29] (Fig. 1). First, the following borders of the outer retina were automatically detected by Spectralis HRA system: outer retinal boundary corresponding to BrM and RPE boundary corresponding to an interpolation of outer and inner RPE border in all SD-OCT scans (37 B-scans, scan area 20° × 15° (6.8 × 4.4 mm), distance between B-scans 121 μm). In addition, a manual correction of those boundaries was performed by two graders (VS and LA) for more accurate results [30]. Then, a circular 6-mm grid was placed manually on the fovea in order to calculate the DV in all 37 B-scans (Fig. 2). Although ETDRS grid area is slightly greater than the area of 37 B-scan (4.4 mm vertically), the measured area was equal for all eyes.

### Sample collection and complement measurement

Concentrations of the following complement factors and proteins were assessed: complement proteins 3 (C3), 4 (C4), 5 (C5); activation products of complement factor 3 (C3a) and Ba, C3b/iC3b; complement factors B, D, H, I (CFB, CFD, CFH, CFI); and total protein. Methods of collection and measurement are elaborated in detail by Altay et al. [18]. Briefly, undiluted AH samples were collected before any ophthalmic surgery at the beginning of the cataract operation and were stored at − 80 °C for analysis. Concentrations and quantifications for the different complement and total protein were performed according to the manufacturer’s instructions using MicroVue-plus ELISA, Milliplex Human Complement Panels 1 and 2, Luminex, Quant-iT Protein Assay Kit.

### Statistical analysis

All statistical tests were performed using SPSS software version 25.0 (SPSS Statistics, Version 25.0. Armonk, NY: IBM Corporation) at a significance level of *P* < 0.05. Pearson’s chi^2^ test was used for categorical variables and Mann-Whitney *U* test for continuous variables for comparison between controls and AMD patients. Spearman correlation test was used for correlations between imaging biomarkers and complement factors among all subjects. A partial non-parametric correlation test controlled for the parameters age and gender separately was used to confirm significant correlations between imaging biomarkers and complement factors.

## Results

The analyses of 106 eyes included 71 controls and 35 eyes with AMD of which 14 had early and 21 had intermediate AMD. Table 1 shows epidemiological and baseline data.

**Table 1:** Demographic data of all patients

	No AMD (*N* = 71)	Early/intermediate AMD* (*N* = 35)	*P* value
Age years (mean ± SD)	70.60 ± 8.31	79.23 ± 5.78	*< 0.001*
Male gender, *n* (%)	32/71 (45.1%)	20/35 (57.1%)	0.242
BCVA Logmar, (mean ± SD)	0.38 ± 0.37	0.35 ± 0.32	0.746
Axial length, (mean ± SD)	23.65 ± 1.28	23.79 ± 1.27	0.940
Hypertension, *n* (%)	40/ 68 (58.8%)	19/31 (61.3%)	0.817
Diabetes, *n* (%)	14/ 71 (19.7%)	5/ 35 (14.3%)	0.493
Glaucoma, *n* (%)	29/ 70 (41.4%)	11/33 (33.3%)	0.733
Total protein ng/ml (median)	490.50	525.50	0.176
C3 ng/ml (median)	504.82	936.34	*0.017*
C3a ng/ml (median)	3.32	4.01	*0.026*
C3b/i C3b ng/ml (median)	529.00	1011.50	*0.032*
Ba ng/ml (median)	6.93	8.88	*0.003*
C4 ng/ml (median)	291.64	253.75	0.685
C5 ng/ml (median)	134.00	1158.80	*0.006*
CFB ng/ml (median)	259.63	359.84	*0.039*
CFD ng/ml (median)	81.07	93.95	0.924
CFH ng/ml (median)	44.89	55.95	0.238
CFI ng/ml (median)	83.80	106.00	0.368

Pearson’s chi^2^ test for categorical variables; Mann-Whitney *U* test for continuous variables

*Early/intermediate AMD patients without any geographic atrophy and without choroidal neovascularization

*AMD*, age-related macular degeneration; *BCVA*, best correlated visual acuity; complement factors 3a (C3a), Ba, C3b/iC3b; complement factors B, D, H, and I (CFB, CFD, CFH, CFI); complement proteins 3 (C3), 4 (C4), 5 (C5); *SD*, standard deviation

Age differed significantly among the groups, while gender, best correlated visual acuity, axial length, hypertension, diabetes, and glaucoma did not. Overall, age correlated with C3b/iC3b (*r* = 0.288; *P* = 0.031), Ba (*r* = 0.312; *P* = 0.002), CFD (*r* = 0.249; *P* = 0.013), and CFI (*r* = 0.217; *P* = 0.033). Gender correlated with total protein (*r* = − 0.458; *P* < 0.001), C3b/iC3b (*r* = − 0.320; *P* = 0.016), Ba (*r* = − 0.201; *P* = 0.045), C4 (*r* = − 0.297, *P* = 0.003), CFB (*r* = − 0.257; *P* = 0.010), CFD (*r* = − 0.297; *P* = 0.003), CFH (*r* = − 0.352; *P* < 0.001), and CFI (*r* = − 0.361, *P* = < 0.001).

Complement components C3, C3a, C3b/iC3b, Ba, C5, and CFB levels were significantly upregulated within the early/intermediate AMD group in comparison with controls (*P* for all ≤ 0.05, Table 1). Total protein levels were not different between the two groups (*P* = 0.176, Table 1).

Eleven eyes (31.4%) in the AMD group had RPD and twelve eyes (34.3%) had HRF. Seven cases (20.0%) had simultaneously HRF and RPD. Total DV in the AMD groups was in average 0.41 ± 0.15 mm^3^. None of the cases in the control group had any RPD, HRF, or drusen.

DV correlated significantly with C3b/iC3b (*r* = 0.285; *P* = 0.034), Ba (*r* = 0.262; *P* = 0.009), and C5 (*r* = 0.430; *P* = 0.005), and showed a tendency towards correlation with C3a (*r* = 0.198; *P* = 0.057).

After controlling for age, the correlation between DV and C3a (*r* = 0.226; *P* = 0.031) as well as the correlation between DV and C5 (*r* = 0.441; *P* = 0.004) remained statistically significant. Also, after controlling for gender, correlations between DV and Ba (*r* = 0.251; *P* = 0.012), C5 (*r* = 0.495; *P* = 0.001), and C3b/iC3b (*r* = 0.294; *P* = 0.030) remained significant, as well as a tendency towards C3a (*r* = 0.199; *P* = 0.057).

HRF showed a significant correlation with C5 (*r* = 0.388; *P* = 0.011) and RPD showed a slight tendency towards a correlation with CFB (*r* = 0.196; *P* = 0.050). The correlation of HRF with C5 remained significant after controlling for age (*r* = 0.384; *P* = 0.013) and gender (*r* = 0.401; *P* = 0.009). The correlation of RPD with CFB remained significant after controlling for gender (*r* = 0.239; *P* = 0.017), but not after controlling for age (*r* = 0.187; *P* = 0.064).

Table 2 provides a summary of the correlation between imaging parameters and complement activation levels.

**Table 2:** Correlation between imaging biomarkers and complement factors

	*N*	Drusen volume		Reticular pseudodrusen		Hyperreflective foci	
		*P* value	Correlation coefficient	*P* value	Correlation coefficient	*P* value	Correlation coefficient
Total protein	94	0.226	0.126	0.364	0.095	0.761	0.032
C3	98	0.077	0.180	0.257	0.116	0.377	0.090
C3a	93	*0.057*	0.198	0.210	0.131	0.380	0.092
C3b/i C3b	56	**0.034**	0.285	0.152	0.194	0.395	0.116
Ba	100	*0.009*	0.262	0.090	0.170	0.087	0.172
C4	99	0.931	0.009	0.238	0.120	0.551	0.061
C5	42	*0.005*	0.430	0.133	0.236	*0.011*	0.388
CFB	100	0.115	0.159	*0.050*	0.196	0.103	0.164
CFD	99	0.935	− 0.008	0.362	0.093	0.758	0.031
CFH	90	0.273	0.117	0.106	0.171	0.142	0.156
CFI	96	0.451	0.078	0.135	0.154	0.567	0.059

Complement factors 3a (C3a), Ba, C3b/iC3b; complement factors B, D, H, and I (CFB, CFD, CFH, CFI); complement proteins 3 (C3), 4 (C4), 5 (C5); *CI*, confidence interval; *OR*, odds ratio

## Discussion

This study evaluated the association between local complement activation and DV, HRF, and RPD as imaging biomarkers for AMD progression in patients with early and intermediate AMD. DV correlated significantly with C3b/iC3b, Ba, and C5 and showed a slight tendency towards a correlation with C3a, while HRF showed a significant correlation with C5, and a tendency towards correlation with Ba. RPD showed only a slight correlation towards CFB, whereas none of the other complement factors was significant. Our findings indicate a disturbed complement regulation in early stages of the disease. Thus, imaging biomarkers can be useful to identify suitable patients in clinical trials for future complement-modulating interventions.

The complement system plays an important role in the response to inflammation in the human body. As an immune privileged organ, the eye requires low-level complement activation in order to provide a level of immune tolerance. Thus, complement disruption is implicated in the development of diverse ocular diseases such as AMD, glaucoma, and diabetic retinopathy [31]. The involvement of the complement system in AMD pathogenesis has been widely accepted, but not completely understood. Large genome-wide association studies implicated the role of complement system in the initiation and progression of AMD [8, 9]. Complement component 3 (C3) is the central molecule of complement system and its cleavage results in transformation to C3a and C3b. Further pathways include cleaving of C5 to C5a and C5b, where C5b assembles with C6, C7, C8, and C9 to form a terminal membrane attack complex (MAC) [32]. In addition, several regulators including CFH and CFB control the activity of the complement system.

Many studies have reported altered systemic complement activation in AMD patients. Some suggest that local intraocular complement activity measured in AH may reflect the complex pathogenesis behind AMD more accurately [19, 20]. In recent years, several studies emphasized the importance of local complement activation in AMD. Schick et al. reported an increase of C3a and Ba concentrations in AH of neovascular AMD patients compared with controls [17]. A recent prospective study of our group reported elevated C3 and C3a levels in AH of patients with early AMD stages compared with controls [18]. In this current study, a significant correlation was found between DV C3b/iC3b and Ba and C5, as well as a tendency towards a correlation with C3a, and between HRF and C5, as well as a trend towards Ba. RPD showed only a trend towards correlation with CFB.

The use of SD-OCT allows an easy, non-invasive, reliable, and reproducible assessment of drusen and DV over time, which makes SD-OCT an indispensable tool for monitoring of AMD progression in clinical routine and clinical trials. In this study, we found a correlation between DV and local complement activation. Our findings are in line with prior histological evidence, which demonstrated the presence of C3, C5, and CFH in drusen [5, 6, 33]. The findings also correspond to genetic studies, which showed an association between greater DV and drusen area with higher number of AMD risk alleles in complement genes [34–36]. Several clinical studies suggested using DV as a quantitative tool to assess the risk for AMD progression [23, 37–39].

SD-OCT allows also the identification of further distinct features such as HRFs [27, 37, 40]. HRF are hyperpigmented small well-circumscribed dots in the neurosensory retina and are regarded also as risk factors for AMD progression [21, 22, 41–43]. Furthermore, HRF have also been associated with several known AMD risk alleles [44]. In this study, HRF were associated with local C5 upregulation. The origin of HRF is yet unknown; however, they are thought to be a complex of phagocytized RPE, lipids, and immune cells [40, 45, 46]. The presence of HRF might reflect a degree of local inflammation including complement upregulation in the eye, and therefore, could be suitable for monitoring of the patients.

Interestingly, in this study, RPD showed only slight correlation with CFB and none of the other complement factors. Like DV and HRF, RPD have been also recognized as a risk factor for progression to advanced AMD [23, 24, 47, 48]. Furthermore, the presence of RPD has been associated with major AMD risk polymorphisms in *ARMS2/HTRA1* genes [24, 49, 50]. RPD are located in the subretinal space and share compositional similarities with conventional drusen, yet their lipid composition is different than soft drusen [51–55]. Nevertheless, RPD appear also in other retinal diseases such as Sorsby fundus dystrophy and pseudoxanthoma elasticum, and a common pathogenetic pathway involving BrM and RPE interface is possible [56]. In a recent study, RPD patients were reported to have different AH protein profiles in comparison with patients with soft drusen [55]. In the same study, it was speculated that RPD formation is linked to a dysfunctional RPE, which secretes proteins aberrantly towards the apical surface instead of onto basolateral surface leading to the accumulation of deposits in subretinal space [55].

Another explanation for the divergence between drusen and RPD in correlation with local complement activation might be related to their different anatomic proximity to BrM. The BrM, located between retinal pigment epithelium and choroid, separates the retina strategically from the general circulation [57]. Beside lipids, it also accumulates local complement regulators such as complement factor H-like protein 1 and complement factor H-like protein 4 [58–60]. Disruptions in BrM are most likely to facilitate local complement imbalance, leading to lipid accumulation between RPE and BrM and ultimately to drusen formation [59]. Therefore, it is plausible to think that drusen most accurately reflect the local complement dysregulation in BrM, while RPD appear rather as a RPE dysfunction.

Therapeutic approaches aiming at modulation of the complement system are gaining importance in different eye diseases. In glaucoma, experimental studies suggest a protective effect after complement modulation therapies [61, 62]. In AMD, Phase II studies reported promising results, yet until now, none of the phase III trials has been successful [63]. Currently, APL-2, a complement component 3 inhibitor, is tested in a phase III trial to assess its potential to reduce the risk of progression of geographic atrophy [63]. Analysis of complement activation in AH of patients with other eye diseases such as glaucoma might highlight common pathways and contribute to novel drug developments.

Correlation of DV and HRF with different complement factors in AH suggests a high turnover of local complement activation in early stages before converting to advance AMD. Therapeutic complement modulation in earlier AMD stages before the onset of irreversible tissue damage may lead to a better treatment success [16]. Yet, for this extensive approach, selection of suitable patients, depending on their genetic profile and local complement activation level could be helpful. Our preliminary results indicate that patients with high DV and presence of HRF could be better suited to participate in such research. Despite showing statistical significance, the correlation coefficients in this study mostly indicated fair or poor correlation levels. These results should be validated in larger studies, especially as stratification would probably allow identifying subgroups or patterns with stronger correlation of imaging data and complement activation. Further limitations include the relatively small sample size, a lack of genetic profile, systemic complement measurements, and automated algorithm for more exact calibration of the grading results. A better understanding of the complex interaction between complement system, imaging biomarkers, and genetic risk factors could be helpful for developing individualized AMD treatment decisions [34].

To minimize confounding effects, patients with systemic/current immunosuppressive therapy, as well as patients suffering from systemic diseases associated with possible complement activation, were not included in this analysis. These strict exclusion criteria might have caused a selection bias. Moreover, this pilot study included relatively small sample size limiting the possibility for multivariate analysis. Furthermore, smoking status was not available as information for the subjects, which may also have an influence on the complement milieu. It remains also possible that subclinical nonexudative macular neovascularization were included, since OCT-angiography imaging was not available in our cohort. DV was only assessed by SD-OCT. Also, no longitudinal data were available to assess the dynamic of DV and RPD over time. An automated algorithm using color fundus photographs and SD-OCT data via artificial intelligence could yield an efficient tool for future studies.

Analysis of associations between distinct AMD phenotypes and local complement activation represents an important and innovative approach in AMD research. Our study contributes by providing data on early AMD and complement activation. Implementation of additional information about systemic complement activation and genetic profile of those patients might be valuable for future studies. To conclude, HRF and drusen parameters but not RPD correlate with local complement activation in in early AMD patients’ AH. Imaging biomarkers could be useful to identify suitable patients for future clinical trials with complement-modulating therapies.

## Data Availability

All data and material are property of University of Cologne.
